# Open-label randomized control trial between low pressure support and T-piece method for discontinuation from mechanical ventilator and extubation in general surgical ICUs

**DOI:** 10.1186/cc14348

**Published:** 2015-03-16

**Authors:** K Chittawatanarat, S Orrapin, S Orrapin

**Affiliations:** 1 Chiang Mai University, Chiang Mai, Thailand

## Introduction

In routine practice for most surgical patients in Thailand, all appropriated patients with planned discontinuation of mechanical ventilator (MV) are routinely changed to T-piece before extubation. However, this method needs to alter the instrument for testing tolerability of the patient. The objective of this study was to compare continuous low pressure support (PSV) and the T-piece method (T) before discontinue mechanical ventilation and extubation in the surgical intensive care unit (SICU).

## Methods

We performed a prospective open-label randomized control study (non-inferiority trial) in SICU patients who were intubated and used mechanical ventilation, and appropriated discontinuation of the ventilator between June 2011 and November 2013. All patients underwent the same weaning protocol. The appropriated patients for discontinuation of MV were randomized into low pressure support up to 7 cmH_2_O and T-piece method. Reintubation within 72 hours, pneumonia after extubation, and hospital mortality were recorded. The statistical significant difference was considered when *P *< 0.05.

## Results

A total of 520 patients were randomized into two groups: low pressure support group (260 patients) and T-piece group (260 patients). There was no difference in age, gender, body mass index, comorbidity, site of surgery, Charlson Comorbidity Index and Acute Physiologic and Chronic Health II score between groups (*P >*0.05). Regarding the intention to treat analysis, there were no differences between groups in reintubation rate (PSV 10% vs. T 14.6%; *P *= 0.109), pneumonia after extubation (PSV 14.2% vs. T 11.9%; *P *= 0.435) and hospital mortality rate (PSV 3.1% vs. T 3.5%; *P *= 0.805).

## Conclusion

The outcomes after discontinuation of the mechanical ventilator between low pressure support and T-piece was not difference in term of reintubation, pneumonia after extubation and hospital mortality.
